# COVID-19 Acute Respiratory Distress Syndrome: Treatment with Helmet CPAP in Respiratory Intermediate Care Unit by Pulmonologists in the Three Italian Pandemic Waves

**DOI:** 10.3390/arm91050030

**Published:** 2023-09-20

**Authors:** Martina Piluso, Clarissa Ferrari, Silvia Pagani, Pierfranco Usai, Stefania Raschi, Luca Parachini, Elisa Oggionni, Chiara Melacini, Francesca D’Arcangelo, Roberta Cattaneo, Cristiano Bonacina, Monica Bernareggi, Serena Bencini, Marta Nadalin, Mara Borelli, Roberto Bellini, Maria Chiara Salandini, Paolo Scarpazza

**Affiliations:** 1 Lung Unit, Cardiothoracic Vascular Department, Vimercate Hospital, 20871 Vimercate, Italy; martina.piluso@asst-brianza.it (M.P.); pierfranco.usai@asst-brianza.it (P.U.); stefania.raschi@asst-brianza.it (S.R.); luca.parachini@asst-brianza.it (L.P.); elisa.oggionni@asst-brianza.it (E.O.); chiara.melacini@asst-brianza.it (C.M.); francesca.darcangelo@asst-brianza.it (F.D.); roberta.cattaneo@asst-brianza.it (R.C.); cristiano.bonacina@asst-brianza.it (C.B.); monica.bernareggi@asst-brianza.it (M.B.); serena.bencini@asst-brianza.it (S.B.); roberto.bellini@asst-brianza.it (R.B.); mariachiara.salandini@asst-brianza.it (M.C.S.); paolo.scarpazza@asst-brianza.it (P.S.); 2 Research and Clinical Trials Office, Poliambulanza Foundation Hospital, 25124 Brescia, Italy; claclafer@gmail.com; 3 School of Medicine and Surgery, University of Milano-Bicocca, 20126 Milan, Italy; maartanadalin@gmail.com (M.N.); maraborelli123@gmail.com (M.B.); 4 Cardiothoracic Vascular Department, Respiratory Unit, Fondazione IRCCS San Gerardo dei Tintori, 20900 Monza, Italy

**Keywords:** COVID-19 ARDS, Helmet CPAP, RICU, corticosteroids, pulmonologist

## Abstract

**Highlights:**

**What are the main findings?**

- Good clinical outcomes with H-CPAP in RICU, especially in mild and moderate CARDS.
- Significant improvement of prognosis in the three different waves: patients’ diseases were found to be progressively slightly less severe. (No patient had yet received at least one dose of vaccination against COVID-19.)

**What is the implication of the main finding?**

- H-CPAP success strongly correlates with worst PaO_2_/FiO_2_ ratio and D-dimer level at admission.
- Relevance of proper management during hospitalization by pulmonologists in RICU.

**Abstract:**

COVID-19 Acute Respiratory Distress Syndrome (CARDS) is the most serious complication of COVID-19. The SARS-CoV-2 outbreaks rapidly saturated intensive care unit (ICU), forcing the application of non-invasive respiratory support (NIRS) in respiratory intermediate care unit (RICU). The primary aim of this study is to compare the patients’ clinical characteristics and outcomes (Helmet-Continuous Positive Airway Pressure (H-CPAP) success/failure and survival/death). The secondary aim is to evaluate and detect the main predictors of H-CPAP success and survival/death. A total of 515 patients were enrolled in our observational prospective study based on CARDS developed in RICU during the three Italian pandemic waves. All selected patients were treated with H-CPAP. The worst ratio of arterial partial pressure of oxygen (PaO_2_) and fraction of inspired oxygen (FiO_2_) PaO_2_/FiO_2_ during H-CPAP stratified the subjects into mild, moderate and severe CARDS. H-CPAP success has increased during the three waves (62%, 69% and 77%, respectively) and the mortality rate has decreased (28%, 21% and 13%). H-CPAP success/failure and survival/death were related to the PaO_2_/FiO_2_ (worst score) ratio in H-CPAP and to steroids’ administration. D-dimer at admission, FiO_2_ and positive end expiratory pressure (PEEP) were also associated with H-CPAP success. Our study suggests good outcomes with H-CPAP in CARDS in RICU. A widespread use of steroids could play a role.

## 1. Introduction

Acute hypoxemic respiratory failure (AHRF) is the most common cause of intensive care unit (ICU) admission in adult patients, often leading to endotracheal intubation and invasive mechanical ventilation (IMV). Although COVID-19 causes very mild symptoms in most cases, approximately 20% of the patients develop acute hypoxemic respiratory failure (AHRF) with bilateral interstitial pneumonia [1]. Acute respiratory distress syndrome (ARDS) is the most serious complication of COVID-19 that occurs in 20–41% of patients with AHRF [2]. Despite the progress achieved in supportive care, the mortality rate of ARDS in ICU is still high (35–40%) and it increases with the severity of hypoxemia (27% in mild, 32% in moderate, 45% in severe ARDS, as defined by the Berlin Definition) [3].

In COVID-19 acute respiratory distress syndrome (CARDS) treated with IMV in ICU, prognosis seems to be even worse than that associated with non-COVID-19-related ARDS, and it varies widely [4,5,6,7]. In Lombardy, northern Italy, the COVID-19 pandemic has led to a substantial increase in the number of patients admitted to hospital with CARDS. In particular, in the first wave, this produced a heavy burden on the healthcare system, especially on ICUs, which easily ran out of resources since almost 10% of the hospitalized COVID-19 patients needed IMV. Until the outbreak of COVID-19 pandemic, evidence suggested the limiting of non-invasive respiratory support (NIRS) to carefully selected patients with mild-to-moderate ARDS and to apply it in experienced centers with close monitoring of blood gases and respiratory mechanics in order to avoid delayed intubation in case of failure [8,9].

The frequent lack of ICU beds has pushed authorities to create respiratory intermediate care units (RICU) in order to face the increasing number of patients with CARDS who need respiratory support and monitoring [10]. This can be carried out by pulmonologists with good previous experience in treating severe community-acquired pneumonia with helmet continuous positive airway pressure (H-CPAP) [11] where H-CPAP had previously demonstrated good efficacy [11,12]. Concerning NIRS, CPAP was significantly associated with a lower risk of mortality [13,14,15], and the H-CPAP has been proposed as an alternative to facemask [16]. In addition, for healthcare workers’ protection from SARS-CoV-2 infection, the helmet has negligible air dispersion [17]. Applying a single level of pressure during the entire respiratory cycle, CPAP enables a reduction in the risk of excessive transpulmonary pressure and contributes to lung protection (reducing the risk of patient self-induced lung injury (P-SILI)). In addition, H-CPAP was available for all treated patients and it was easier to manage than pressure support ventilation (PSV) with Helmet. Other forms of NIRS, such as high flow nasal cannula (HFNC), were numerically unavailable.

The primary aim of this study is to compare the patients’ clinical characteristics and outcomes (H-CPAP success defined as direct discharge from RICU without intubation and survival/death) during the three different waves. The secondary aim is to evaluate and detect the main predictors of H-CPAP success and survival/death in patients selected according to CARDS criteria.

## 2. Materials and Methods

### 2.1. Sample Selection

During the three waves of COVID-19 pandemic, 2159 patients with COVID-19 pneumonia, defined as the presence of interstitial pulmonary infiltrates and a positive SARS-CoV-2 nasal-pharyngeal swab, were admitted at Vimercate Hospital, Lombardy, Italy between March 2020 and May 2021. Of these, 871 patients were hospitalized in the Pulmonology Division. Among these, 515 were enrolled in our observational prospective study based on the development of CARDS defined by the Berlin Definition (ratio of arterial partial pressure of oxygen (PaO_2_) and fraction of inspired oxygen (FiO_2_) PaO_2_/FiO_2_ ≤ 300 with positive end-expiratory pressure (PEEP) ≥ 5 cm H_2_O and bilateral interstitial pneumonia) during hospital stay [3]. The exclusion criteria were as follows: age higher than 81 (patients older than 81 were rarely admitted to ICU, and there were none in our case study) and patients who did not develop CARDS during hospitalization. All selected patients (ages 18–80) were treated with H-CPAP in RICU. No patient had yet received one dose of vaccination against COVID-19.

### 2.2. Clinical Procedures and Monitoring

In our hospital, the ad hoc RICU dedicated to COVID-19 patients with AHRF (implemented from 6 to 50 beds) was characterized by continuous multi-parametric monitors, access to high-flow oxygen and air sources with systems to obtain adequate values of delivered FiO_2_, onsite life support and intubation kit, a nurse patient ratio between 1:6 and 1:10, and full day shifts run by pulmonologists.

All patients included in the study were hemodynamically stable, with a normal Glasgow Coma Scale (GCS) score, (GCS = 15) and did not show multi-organ system failure, acidosis or hypercapnia [18]. They poorly responded to treatment with high-flow oxygen therapy with a Venturi mask or a non-rebreathing oxygen mask (oxygen saturation (SpO_2_) ≤ 92%, respiratory rate > 24, Breaths Per Minute, thoraco-abdominal dyssynchrony).

H-CPAP was delivered with a pressure between 5 and 15 cm H_2_O. and FiO_2_ between 50 and 100%, with a target oxygen saturation of 92–98%; if reducing the FiO_2_ up to 50%, the saturation remained above 98%, and the PEEP also progressively decreased. During H-CPAP therapy, the patients were moved, when feasible, into prone position, which was maintained for at least two hours. After two hours, the blood gas control PaO_2_/FiO_2_ ratio was re-calculated. The most critical patients were selected by pulmonologists and evaluated by intensivists to decide on ICU transfer.

The indication for IMV included the following criteria: (1) a reduced level of consciousness, (2) persistent hypoxemia with altered mechanical breathing, (3) H-CPAP intolerance, (4) hemodynamic instability, and (5) multi-organ failure.

The Do-Not-Intubate (DNI) order was the decision to withhold intubation and to use H-CPAP as the “ceiling” treatment considering the patient’s characteristics and the reduced availability of ICU beds. DNI criteria was considered by intensivists only in cases in which intubation was necessary, not at the admission stage.

Unless contraindicated, prophylactic low-molecular-weight heparin was administered to all patients, except those already on home anticoagulation therapy. In the first wave, Computed Tomography (CT) Angiography (Revolution 128S, General Electric Company, Boston, MA, USA) was performed as soon as the clinical condition worsened in association with a significant increase in D-dimer (immunoturbidimetric method - Instrument: ACLTOP550, Instrumentation Laboratory, Bedford, MA, USA; Reagent: Instrumentation Laboratory, Bedford, MA, USA). With D-dimer >1000 and CT Angiography negative for pulmonary embolism, 100 units/kg of low-molecular-weight heparin was administered daily [19]. In the second and third waves, the D-dimer dosage was performed daily for the first week of hospital stay and, in case of a significant increase even without clinical worsening, CT Angiography was performed. Therapeutic low-molecular-weight heparin (100 unit/kg twice a day) was administered in case of confirmed pulmonary embolism.

In the first wave, most patients were treated with systemic corticosteroids (methylprednisolone at a dose of 1 mg/kg per day) for 10 days gradually reduced in case of positive outcome. In the second and third waves, all patients received corticosteroids.

### 2.3. Statistical Analysis

Descriptive statistics for the socio-demographic, clinical and outcome variables are given in terms of mean, median and standard deviation for numerical variables and percentage distribution for categorical variables. Normality assumption was assessed using the Shapiro–Wilk test and the graphical inspection via QQ-plots. ANOVA or corresponding nonparametric Kruskal–Wallis tests were applied for comparing numerical variables across three categories of pandemic waves. Chi-squared tests were applied for analyzing the association between categorical variables and waves.

Associations among socio-demographic and clinical variables with the three outcomes (one at a time) were evaluated using univariate logistic regression models. Subsequently, multiple logistic models, including, as covariates and factors, all the significant variables detected through univariate models, were performed. The choice of the best model, in terms of significant predictors which better explain the outcome, was carried out by following the stepwise procedure [20].

Finally, the survival analysis through the Kaplan–Meier (KM) curve was used to analyze the survival time in hospital. Differences in KM-curves between different groups were evaluated using the Log-rank test.

The analyses were carried out using SPSS Statistics for Windows, (Version 26.0) and the software R (R Core Team (2020), https://www.R-project.org/ (accessed on 28 February 2022)). The stepwise procedure was performed using the stepAIC function of the R library MASS. Statistical significance was set at level 0.05.

## 3. Results

### 3.1. Socio-Demographic, Clinical and Outcomes Assessments across the Three Waves

Sample characteristics and features’ descriptions of this prospective observational study are reported in Table 1, Table 2 and Table 3. The sample did not show differences among waves for the socio-demographic features except for the categorical variable ‘smoke’. With regard to the comorbidities, differences among waves were found for patients with diabetes, tumor and chronic renal failure: a higher percentage of patients with such comorbidities were detected in the second and the third waves.

Regarding the pharmacological treatments, there was a reduction in the administration of antivirals, tocilizumab and hydroxychloroquine over the course of the waves, while there was an increase in the administration of azithromycin and steroids. Remdesivir was rarely used in all waves (only in 11 patients, 2%).

Additionally, the choice to prone patients increased significantly over the course of the waves.

D-dimer test was higher in wave 1 with respect to wave 2 and 3, while interleukin-6 (IL-6 worst) was lower in wave 3 with respect to the previous waves. Interleukin-6 was dosed using the sandwich electrochemiluminescence immunoassay method (ECLIA) (by ACLTOP550, Instrumentation Laboratory, Bedford, MA, USA; Reagent: Instrumentation Laboratory, Bedford, MA, USA). Concerning H-CPAP treatment, PEEP and FiO_2_ were statistically higher in wave 1 with respect to the subsequent waves, whereas PaO_2_/FiO_2_ in oxygen and PaO_2_/FiO_2_ (worst) were significantly lower in the first wave with respect to waves 2 and 3.

General improvements over the course of the waves have also been noticed for the main outcomes: mortality, H-CPAP success and DNI. In particular, the mortality rate was, respectively, 28% in the first wave, 20.5% in the second and 12.9% in the third wave. H-CPAP success increased from 62% (93/150) to 69.4% (125/180) and to 78.4% (142/185), and DNI decreased from 22 (14.6%) to 16 (8.8%) and to 7 (3.8%) (Table 1, Table 2 and Table 3).

**Figure 1:**
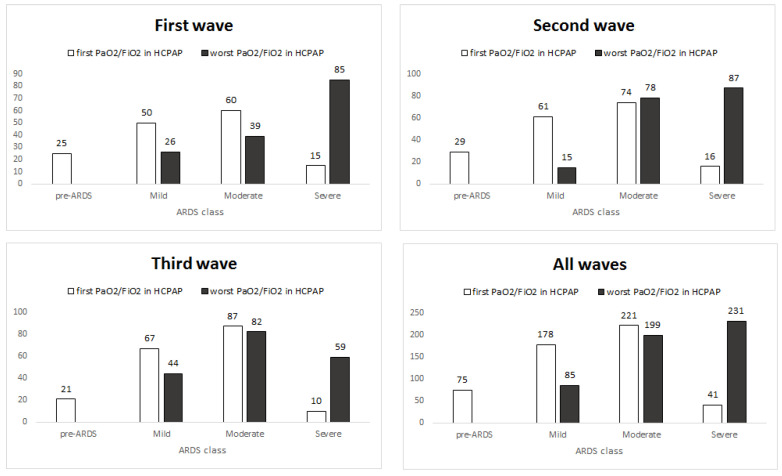
CARDS classes at different waves: number of patients by CARDS class for the parameter PaO_2_/FiO_2_ taken at two time points (first and worst in H-CPAP).

Most patients at admission presented a CARDS pattern. CARDS was mild in 178 (34.6%), moderate in 221 (42.9%) and severe in 41 (7.9%). There are a number of patients (*n* = 75, 14.6%) who did not fulfill CARDS criteria at admission (pre-CARDS) but all of them developed CARDS during hospital stay (Figure 1). The worst PaO_2_/FiO_2_ ratio during H-CPAP stratified the subjects into mild (82–15.9%), moderate (202–39.2%) and severe (231–44.9%) CARDS (Figure 1).

### 3.2. Complications

Among the 515 patients, 360 (70%) were successfully discharged without IMV; 104 were transferred to ICU to receive IMV and, of these, 52 finally survived. A total of 45 received the DNI order. Of the 53 patients who died in the RICU ward, 43 had DNI orders, 1 acute myocardial infarction, 2 massive pulmonary embolism, 3 cardio-circulatory arrests, 1 stroke in paroxysmal atrial fibrillation, 1 respiratory arrest and 2 sepsis after ICU discharge.

The main complications (Table S1) that developed during hospital stays included the following: 54 pulmonary embolism, 8 thrombosis, 10 bleedings, 15 pneumomediastinum, 5 pneumothoraxes, 15 supraventricular tackyarrhythmias, and 12 severe bacterial superinfections. The rate of patients with pulmonary embolism (on the total number of patients) was statistically different across the waves (*p*-values < 0.001), as well as the rate of deaths with pulmonary embolisms on the total of deaths (*p*-values = 0.033).

### 3.3. Predictors of the H-CPAP Success and Survival/Death Outcomes

For each of the two outcomes, the most prominent predictors are reported in Tables S2 and S3 in the Supplementary Materials.

Considering all the statistically significant variables, multiple logistic models (with stepwise procedure for the variable selections) were applied in order to obtain the best predictors of each outcome. The results are reported in Table 4. The most important factors for the H-CPAP success were as follows: the worst PaO_2_/FiO_2_ ratio during H-CPAP and FiO_2_ (both with *p*-values < 0.001), as high levels of PaO_2_/FiO_2_ and FiO_2_ were associated with better (Odds Ratio (OR) = 1.038) and worse (OR = 0.944) H-CPAP success, respectively. Additionally, the administration of steroids had a relevant impact (*p*-values = 0.001) on H-CPAP success: the administration of steroids increases the probability of H-CPAP success by almost 14 times with respect to non-administration (OR = 13.92). In addition, D-dimer at admission and the level of PEEP were also found to be significantly associated with H-CPAP success: an increase of 1000 unit in D-dimer level reduced H-CPAP success by 9.5% (OR = 0.905), while an increase of 1 unit in PEEP decreased the probability of H-CPAP success by about 12% (OR = 0.88). Regarding survival/death, the worst PaO_2_/FiO_2_ ratio during H-CPAP and the administration of steroids were also the best predictors for this second outcome: a high level of PaO_2_/FiO_2_ was associated with a lower probability of death (OR = 0.96), while patients treated with steroids showed a lower probability (of about 77%: OR = 0.23) of death with respect to patients who did not undergo steroid therapy.

### 3.4. Survival Analysis

**Figure 2:**
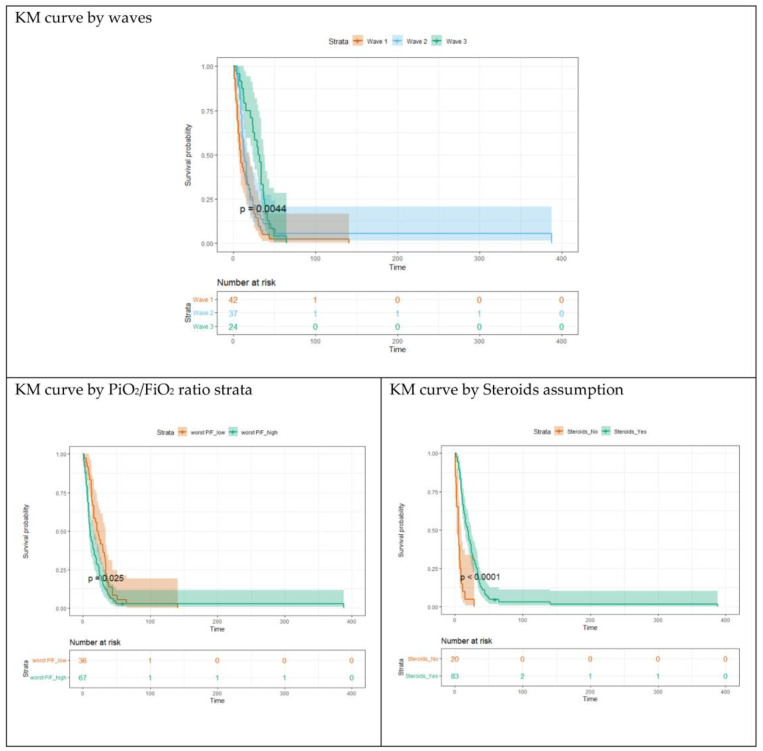
Survival analysis output: Kaplan–Meier survival curves.

An exhaustive survival analysis was carried out by considering the waves and the predictors of the two outcomes highlighted in the previous analyses as predictors (groups) of the KM curves. In Figure 2, KM curves for the three waves are depicted. Interestingly, the curves for waves 1 and 2 are not statistically different (Log rank test *p*-values = 0.196), while the curve for wave 3 is different from the previous ones (*p*-values = 0.004). KM curves were also estimated for different levels of worst PaO_2_/FiO_2_ ratio during H-CPAP (less than first quartile = 77; ≥77), and for steroid therapy (yes vs. no). All these factors were statistically associated with survival and all the KM curves were statistically different among the predictor groups (*p*-values < 0.025 for worst PaO_2_/FiO_2_; *p*-values < 0.001 for steroid therapy).

## 4. Discussion

This prospective observational study aims to better understand the effectiveness of H-CPAP in patients who developed CARDS during hospitalization in RICU.

The patient samples did not show differences among waves for socio-demographic features (Table 1). In the first wave, less compromised patients (fewer patients with diabetes and chronic renal failure and fewer smokers) presented a worse trend. During the second and third pandemic waves, the hospital mortality for patients admitted with CARDS was significantly reduced compared to that registered in the first pandemic period. H-CPAP success increased and DNI numbers decreased.

In the first wave, most patients were treated with systemic corticosteroids. In contrast, in the second and third waves, practically all patients received corticosteroids following new clinical trials [21] and our previous experience with steroid use in severe community-acquired pneumonia [11,22]. In the first wave, PaO_2_/FiO_2_ in oxygen was significantly lower and D-dimer significantly higher. These were demonstrated to be independent risk factors for adverse outcomes [23,24] and the result is also confirmed in our study. In addition, D-dimer at admission, worst D-dimer and worst IL-6 were significantly higher in the first wave, suggesting more severe inflammation. Worst PaO_2_/FiO_2_ in H-CPAP was significantly lower in the first wave. No differences were found between the first PaO_2_/FiO_2_ in H-CPAP among the three waves and the patients with preARDS and mild, moderate, and severe ARDS at admission were equally distributed during the different waves; however, this parameter was obtained with PEEP progressively decreasing.

Helmet success and survival/death outcomes progressively improved over the course of the three waves reflecting a slight progressive reduction in patient severity associated with improved clinical management (practically 100% steroid in the second and third waves; daily D-dimer monitoring for pulmonary embolism diagnosis; progressive increase in prone position).

DNI order was considered only in cases that needed intubation and decreased during the three waves due to the higher availability of ICU beds (equal percentage of patients transferred to ICU in the different waves despite higher patient severity in the first period).

CARDS has a biphasic trend confirmed in all three waves (Figure 1). The two stages of the disease correspond to the initially worsening trend of most of our patients, from admission to subsequent days of hospitalization, and they are likely to switch from L (low elastance, low lung weight, low recruitability—ground glass opacities at CT, preserved lung compliance) to H (high elastance, high lung weight, high recruitability—extensive densification at CT) CARDS [25,26,27]. Probably, in the first stage of the disease, improvement in oxygenation through the application of PEEP or pronation is mainly not due to the recruitment, but to the redistribution of perfusion in the lungs [25,28]. In the second stage of the disease, the application of PEEP recruits non-aerated alveoli in dependent pulmonary regions stabilizes the airways and reduces the inhomogeneity of lung volume distribution [18]. PEEP can be applied in spontaneously breathing patients in the form of CPAP [29].

The most important complications are shown in Table S1. The increased frequency of pulmonary embolism diagnosis in the second and third waves is explained by daily D-dimer monitoring and a higher use of CT Angiography.

Low PaO_2_/FiO_2_ ratio during H-CPAP, high FiO_2_ and average helmet PEEP were important factors of H-CPAP failure as a result of more severe AHRF; as already known, the mortality rate of ARDS increases with the severity of hypoxemia [3].

An increase of 1000 unit in D-dimer level (more severe “cytokine storm”) reduces the H-CPAP success by 9.5% [24].

A widespread use of steroids in our center could play a role in good clinical outcomes. Our study shows that the administration of steroids increases the chance to H-CPAP success of almost 14 times, confirming what has been demonstrated in the RECOVERY TRIAL [21], a large multicenter randomized controlled trial where patients receiving dexamethasone had a reduced death rate especially on mechanical ventilation.

Additionally, for the second outcome, survival/death, the worst PaO_2_/FiO_2_ ratio during H-CPAP and the administration of steroids were the best predictors. In our study there is a lower probability of death (77%) with respect to patients who did not undergo steroid therapy (Table 4).

Prone position in non-intubated spontaneously breathing patients is widely applied alongside NIRS. Its effectiveness in reducing intubation rates and mortality and its tolerability, timing and optimal duration are still not completely clear [30]. Prone position has gradually been increased over the course of the three waves based on early suggestions in the literature [30,31]. In our patients, prone position determined a meaningful increase in PaO_2_/FiO_2_ value, although this improvement does not represent a good prognostic factor in itself. This response could give patients a chance to overcome the critical phase of CARDS and avoid intubation. We want to emphasize the fact that, despite the extremely low values of worst PaO_2_/FiO_2_ ratio recorded, 82 (15.9%) mild, 202 (39.2%) moderate, 231 (44.9%) severe CARDS, 70% of our patients were finally discharged without a need for IMV. In mild patients, H-CPAP had a success of 98.8%; in moderate patients, of 93%; and in severe patients, of 41%. In addition, 89 out of 231 patients in the “severe CARDS” group were transferred to ICU and, of these, 44 finally survived, with a final mortality rate of 39.8%, in agreement with the mortality rate described for patients with severe non-COVID-19 ARDS in the ICU (45%). We underline that, in our group of patients, mortality rates in mild and moderate ARDS are inferior to those reported in literature [3,8], considering the different features of patients admitted to ICUs (i.e., multiorgan failure).

Many management models for noninvasive treatment of CARDS in RICU have been proposed in the literature [31,32,33]. To our knowledge, to date, this is the only study entirely carried out in RICU on patients who all presented with CARDS and were all treated with H-CPAP in the three COVID-19 waves. We may therefore assume that the proper management in RICU, the use of H-CPAP as NIRS, prone position, and large steroid use affect the prognosis of patients with CARDS [34].

A constant clinical and parametric monitoring during hospitalization by the pulmonologist in RICU is critical in the prompt recognition and treatment of every possible worsening in clinical conditions, an event than can arise even later in the course of the disease. In fact, the majority of patients moved to a worse CARDS class during hospitalization (Figure 1). Furthermore, our data seem to exclude a possible delay in intubation timing due to H-CPAP treatment and this is remarked by a mortality rate of almost 50% in patients finally admitted to the ICU, substantially comparable with 55% of all Lombardy ICUs [7] and other countries’ experiences [35]. In addition, we must remember that even if delayed intubation is associated with increased mortality in patients with AHRF [35,36], it is also true that premature intubation when NIRS is adequate exposes patients to potentially unnecessary risks associated with IMV [16,37].

Our study has several limitations that can limit the generalizability of our results, including being monocentric, the lack of a control group and the peculiar setting of the study, characterized by an emergency pandemic situation with continuous changes in scientific evidence. Nevertheless, further multicentric trials are needed in order to confirm these data. In addition, the Berlin Definition of ARDS required that patients must be in IMV in moderate and severe ARDS, with the exception of mild ARDS, in which patients can receive CPAP ≥ 5 cm H_2_O. In our study, ARDS was classified as moderate or severe during H-CPAP; however, the new recently published ARDS definition [38] allows for classification as moderate and severe in H-CPAP too.

## 5. Conclusions

Our study suggests good clinical outcomes with H-CPAP in RICU, especially in mild and moderate CARDS.

We observed a significant improvement in prognosis in the three different waves, as the patients’ conditions are found to be progressively slightly less severe.

CARDS has a biphasic trend confirmed in all three waves, with a trend of worsening the patients’ conditions from admission to subsequent days of hospitalization.

The CARDS severity (worst PaO_2_/FiO_2_ in H-CPAP, FiO_2_ in H-CPAP, average PEEP and D-dimer at admission) strongly correlates with the first outcome (H-CPAP success). Worst PaO_2_/FiO_2_ in H-CPAP also strongly correlates with the second outcome (survival/death).

There was a significant prognosis improvement in subjects who received corticosteroids.

Pulmonologists’ proper management during hospitalization in RICU may affect these patients’ trend.

## Figures and Tables

**Table 1:** Socio-demographic features, comorbidity and prognostic score of the whole sample by waves.

Variables	Wave1 Mean [Median] (SD) N = 150	Wave2 Mean [Median] (SD) N = 180	Wave3 Mean [Median] (SD) N = 185	Tot	*p*-Value	Post Hoc
Socio-demographic features						
Age	61.9 [63.3] (10.8)	63.5 [65] (10.9)	62.2 [64] (11.2)	515	0.316	
Sex						
Females	N = 27; 24.3%	N = 38; 34.2%	N = 46; 41.4%	111	0.310	
Males	N = 123; 30.4%	N = 142; 35.1%	N = 139; 34.7%	404		
Body Mass Index (BMI)	29.4 [29.4] (4.7)	30.2 [29] (6.6)	29.7 [29] (4.7)	361	0.847	
Smoke						
No	N = 104; 39.2%	N = 69; 26%	N = 92; 34.7%	265	<0.001	1 vs. 2,3
Yes	N = 1; 4.2%	N = 8; 33.5%	N = 15; 62.5%	24		
Ex-smokers	N = 45; 26%	N= 65; 37.6%	N = 63; 36.4%	173		
Comorbidity and prognostic score						
Hypertension						
No	N = 78; 32.9%	N = 74; 31.2%	N = 85; 35.9%	237	0.142	
Yes	N = 72; 25.9%	N = 106; 38.1%	N = 100; 36%	278		
Ischemic cardiac disease						
No	N = 132; 29.7%	N = 150; 33.8%	N = 163; 36.5%	445	0.349	
Yes	N = 15; 25%	N = 26; 43.3%	N = 19; 31.7%	60		
Cardiovascular disease						
No	N = 129; 30.3%	N = 144; 33.8%	N = 153; 35.9%	426	0.357	
Yes	N = 21; 23.6%	N = 36; 40.4%	N = 32; 36%	89		
Hypercholesterolemia						
No	N = 122; 28.3%	N = 152; 35.3%	N = 157; 36.4%	431	0.646	
Yes	N = 28; 33.3%	N = 28; 33.3%	N = 28; 33.3%	84		
Diabetes						
No	N = 139; 32.7%	N = 136; 32%	N = 150; 35.3%	425	<0.001	1 vs. 2,3
Yes	N = 11; 12.2%	N = 44; 48.9%	N = 35; 38.9%	90		
Neoplasia						
No	N = 144; 31%	N = 149; 32.1%	N = 171; 36.9%	464	<0.001	2 vs. 1,3
Yes	N = 6; 11.8%	N = 31; 60.8%	N = 14; 27.5%	51		
Chronic obstructive pulmonary disease (COPD)/asthma						
No	N = 136; 29.4%	N = 158; 34.2%	N = 168; 36.4%	462	0.571	
Yes	N = 14; 26.4%	N = 22; 41.5%	N = 17; 32.1%	53		
Chronic renal failure						
No	N = 149; 30.5%	N = 164; 33.6%	N = 175; 35.9%	488	0.004	1 vs. 2,3
Yes	N = 1; 3.7%	N = 16; 59.3%	N = 10; 37%	27		
Apache II score	10.6 [10] (3.6)	10.3 [11] (5.0)	9.7 [10] (4.3)	513	0.110	

Percentages are reported by row, i.e., the percentages are expressed in terms of total across waves; percentage by column can be obtained considering the patient numbers by wave reported in the table head. N: number of patients.

**Table 2:** Pharmacological treatment, blood tests, CARDS classes at admission and ICU transfer of the whole sample by waves.

Variables	Wave1 Mean [Median] (SD) N = 150	Wave2 Mean [Median] (SD) N = 180	Wave3 Mean [Median] (SD) N = 185	Tot	*p*-Value	Post Hoc
Pharmacological treatment during hospitalization						
Antivirals						
No	N = 46; 11.3%	N = 176; 43.3%	N = 184; 45.3%	406	<0.001	1 vs. 2,3
Yes	N = 104; 95.4%	N = 4; 3.7%	N = 1; 0.9%	109		
Remdesivir						
No	N = 146; 29%	N = 177; 35.1%	N = 181; 35.9%	504	0.822	
Yes	N = 4; 36.4%	N = 3; 27.3%	N = 4; 36.4%	11		
Azithromycin						
No	N = 113; 43.6%	N = 70; 27.0%	N = 76; 29.3%	259	<0.001	1 vs. 2,3
Yes	N = 37; 14.5%	N = 110; 43%	N = 109; 42.6%	256		
Tocilizumab						
No	N = 139; 27.6%	N= 180; 35.7%	N = 185; 36.7%	504	<0.001	1 vs. 2,3
Yes	N = 11; 100%	N = 0; 0%	N = 0; 0%	11		
Plaquenil						
No	N = 5; 1.4%	N= 180; 48.6%	N = 185; 50%	370	<0.001	1 vs. 2,3
Yes	N = 145; 100%	N = 0; 0%	N = 0; 0%	145		
Steroids						
No	N = 32; 86.5%	N = 4; 10.8%	N = 1; 2.7%	37	<0.001	1 vs. 2,3
Yes	N = 118; 24.7%	N = 176; 36.8%	N = 184; 38.5%	478		
COVID-19 Acute Respiratory Distress Syndrome (CARDS) classes at admission and Intensive Care Unit (ICU) transfer						
CARDS classes (admission)						
Pre-CARDS	N = 25; 33.3%	N = 29; 38.7%	N = 21; 28%	75	0.486	
Mild	N = 50; 28.1%	N = 61; 34.3%	N = 67; 37.6%	178		
Moderate	N = 60; 27.1%	N = 74; 33.5%	N= 87; 39.4%	221		
Severe	N = 15; 36.6%	N = 16; 39%	N = 10; 24.4%	41		
Intensive Care Unit						
No	N = 118; 28.6%	N = 143; 34.7%	N = 151; 36.7%	412	0.777	
Yes	N = 32; 31.1%	N = 37; 35.9%	N = 34; 33%	103		
Blood tests						
D-dimer test (admission) (ng/mL)	3629.8 [630] (9423)	586.6 [296] (1521)	443.2 [288] (919)	502	<0.001	1 vs. 2,3
D-dimer test (worst) (ng/mL)	6939.2 [2067] (12382)	3571.9 [1049] (9468)	2447 [719] (5054)	503	<0.001	1 vs. 2,3
Ferritin (admission) (ng/mL)	1993.3 [1283] (1943)	1894.4 [1027] (2542)	3551.1 [1712] (4384)	186	0.183	
Ferritin (worst)(ng/mL)	2750.4 [1631] (3115)	2368.9 [1274] (2864)	2008.9 [1540] (1498	205	0.290	
Interleukin-6 (admission) (pg/mL)	64.8 [32] (83.4)	60.8 [33] (78.0)	58.8 [34] (73.4)	377	0.925	
Interleukin-6 (worst)(pg/mL)	338.2 [89] (950.5)	171.8 [103.5] (235.7)	113.1 [65.5] (164.5)	398	0.029	2 vs. 3

Percentages are reported by row, i.e., the percentages are expressed in terms of total across waves; percentages by column can be obtained considering the patient numbers by wave reported in the table head. N: number of patients.

**Table 3:** CPAP treatments and outcomes of the whole sample by waves.

Variables	Wave1 Mean [Median] (SD) N = 150	Wave2 Mean [Median] (SD) N = 180	Wave3 Mean [Median] (SD) N = 185	Tot	*p*-Value	Post Hoc
Helmet Continuous Positive Air Pressure (H-CPAP) treatments						
Positive End Expiratory Pressure (PEEP)	12.8 [12] (2.0)	9.7 [9] (7.0)	8.1 [8] (1.2)	512	<0.001	1 vs. 2 vs. 3
FiO_2_	81.3 [80] (12.4)	70.2 [70] (11.6)	71.8 [70] (9.4)	514	<0.001	1 vs. 2,3
PaO_2_/FiO_2_ (in oxygen)	132.3 [115.5] (64.4)	159.6 [137] (53.8)	147.5 [130] (64.0)	514	0.001	1 vs. 2,3
First PaO_2_/FiO_2_ (in H-CPAP)	217.8 [203.5] (105.8)	212.1 [199.5] (91.8)	202.8 [195] (77.4)	515	0.747	
Worst PaO_2_/FiO_2_ (in H-CPAP)	100.5 [80] (56.5)	116.7 [100.5] (53.8)	147.0 [135] (64.5)	477	<0.001	1 vs. 2 vs. 3
PaO_2_/FiO_2_ post-pronation (in H-CPAP)	242.4 [227.5] (119.6)	273.3 [227.5] (108.6)	249.7 [240] (97.1)	396	0.051	
Proned						
No	N = 54; 50.9%	N = 24; 22.6%	N = 28; 26.4%	106	<0.001	1 vs. 2,3
Yes	N = 96; 23.5%	N = 156; 38.1%	N= 157; 38.4%	409		
Do Not Intubate (DNI)					0.002	3 vs. 1,2
No	N = 128; 27.2%	N = 164; 34.9%	N = 178; 37.9%	470		
Yes	N = 22; 48.9%	N = 16; 35.6%	N = 7; 15.6%	45		
Outcomes						
Death						
No	N = 108; 26.2%	N = 143; 34.7%	N = 161; 39.1%	412	0.003	1 vs. 3
Yes	N = 42; 40.8%	N = 37; 35.9%	N = 24; 23.3%	103		
H-CPAP success						
No	N = 57; 36.8%	N = 55; 35.5%	N = 43; 27.7%	155	0.014	1 vs. 3
Yes	N = 93; 25.8%	N = 125; 34.7%	N = 142; 39.4%	360		

Percentages are reported by row, i.e., the percentages are expressed in terms of total across waves; percentages by column can be obtained considering the patient numbers by wave reported in the table head. PEEP: Positive end expiratory pressure; PaO_2_/FiO_2_: ratio of arterial partial pressure of oxygen (PaO_2_) and fraction of inspired oxygen (FiO_2_). N: number of patients.

**Table 4:** Multiple logistic regression models outputs for each of the two outcomes.

Outcome (Dependent) Variable	Independent Variables	Coeff (b)	exp(b) = Odds Ratio (OR) #	Lower Lim OR 95% CI	Upper Lim OR 95% CI	*p*-Value	Nagelkerke R2
H-CPAP success (yes vs. no)	Worst PaO_2_/FiO_2_ (in H-CPAP)	0.037	1.038	1.028	1.048	<0.001	0.54
	FiO_2_ in H-CPAP	−0.057	0.944	0.915	0.974	<0.001	
	Steroids (yes vs. no)	2.63	13.915	2.611	74.207	0.001	
	D-dimer at admission (×1000)	−0.1	0.905	0.980	1	0.031	
	average PEEP	−0.13	0.878	−0.260	−0.001	0.048	
Death (yes vs. no)	Worst PaO_2_/FiO_2_ (in H-CPAP)	−0.038	0.963	0.951	0.974	<0.001	0.41
	Steroids (yes vs. no)	−1.45	0.233	0.075	0.730	0.012	
	Lactate dehydrogenase (LDH) at admission	0.001	1.001	0.999	1.003	0.088	

# OR larger than 1 indicate large probability to belong to H-CPAP success or dead for a unit increase (or to pass from first specified category to second specified reference category) in the independent variable. E.g., an increase of 1 in FiO_2_ in H-CPAP produced a reduced probability (of 1 − 0.944 = 5.6%) of H-CPAP success; similarly, the administration of steroids increases the probability of H-CPAP success by almost 14 times with respect to non-administration. PEEP: Positive end expiratory pressure; PaO_2_/FiO_2_: ratio of arterial partial pressure of oxygen (PaO_2_) and fraction of inspired oxygen (FiO_2_).

## Data Availability

The datasets analyzed during the current study are available from the corresponding author on reasonable request.

## Supplementary Materials

The following supporting information can be downloaded at: https://www.mdpi.com/article/10.3390/arm91050030/s1, Table S1: Complication across the three waves; Table S2: Univariate logistic regression models with H-CPAP success (yes vs. no) as dependent variable (only significant variables/predictors are reported); Table S3: Univariate Logistic regression models with Death (yes vs. no) as dependent variable (only significant variables/predictors are reported).

## References

[1] Ren L.-L., Wang Y.-M., Wu Z.-Q., Xiang Z.-C., Guo L., Xu T., Jiang Y.-Z., Xiong Y., Li Y.-J., Li X.-W. (2020). Identification of a novel coronavirus causing severe pneumonia in human: A descriptive study. Chin. Med. J..

[2] Wang D., Hu B., Hu C., Zhu F., Liu X., Zhang J., Wang B., Xiang H., Cheng Z., Xiong Y. (2020). Clinical Characteristics of 138 Hospitalized Patients With 2019 NovelCoronavirus–Infected Pneumonia in Wuhan, China. JAMA.

[3] Ranieri V.M., Rubenfeld G.D., Thompson B.T., Ferguson N.D., Caldwell E., Fan E., Camporota L., Slutsky A.S. (2012). Acute respiratory distress syndrome: The Berlin Definition. JAMA.

[4] Yang X., Yu Y., Xu J., Shu H., Xia J., Liu H., Wu Y., Zhang L., Yu Z., Fang M. (2020). Clinical course and outcomes of critically ill patients with SARS-CoV-2 pneumonia in Wuhan, China: A single-centered, retrospective, observational study. Lancet Respir. Med..

[5] Bhatraju P.K., Ghassemieh B.J., Nichols M., Kim R., Jerome K.R., Nalla A.K., Greninger A.L., Pipavath S., Wurfel M.M., Evans L. (2020). Covid-19 in Critically Ill Patients in the Seattle Region—Case Series. N. Engl. J. Med..

[6] Richardson S., Hirsch J.S., Narasimhan M., Crawford J.M., McGinn T., Davidson K.W., The Northwell COVID-19 Research Consortium (2020). Presenting Characteristics, Comorbidities, and Outcomes among 5700 Patients Hospitalized with COVID-19 in the New York City Area. JAMA.

[7] Grasselli G., Greco M., Zanella A., Albano G., Antonelli M., Bellani G., Bonanomi E., Cabrini L., Carlesso E., Castelli G. (2020). Risk Factors Associated with Mortality Among Patients with COVID-19 in Intensive Care Units in Lombardy, Italy. JAMA Intern. Med..

[8] Grassi A., Foti G., Laffey J.G., Bellani G. (2017). Noninvasive mechanical ventilation in early acute respiratory distress syndrome. Pol. Arch. Intern. Med..

[9] Demoule A., Hill N., Navalesi P. (2016). Can we prevent intubation in patients with ARDS?. Intensive Care Med..

[10] Grasselli G., Zangrillo A., Zanella A., Antonelli M., Cabrini L., Castelli A., Cereda D., Coluccello A., Foti G., Fumagalli R. (2020). Baseline Characteristics and Outcomes of 1591 Patients Infected with SARS-CoV-2 Admitted to ICUs of the Lombardy Region, Italy. JAMA.

[11] Brambilla A.M., Aliberti S., Prina E., Nicoli F., Del Forno M., Nava S., Ferrari G., Corradi F., Pelosi P., Bignamini A. (2014). Helmet CPAP vs. oxygen therapy in severe hypoxemic respiratory failure due to pneumonia. Intensiv. Care Med..

[12] Cosentini R., Brambilla A.M., Aliberti S., Bignamini A., Nava S., Maffei A., Martinotti R., Tarsia P., Monzani V., Pelosi P. (2010). Faculty Opinions recommendation of Helmet continuous positive airway pressure vs oxygen therapy to improve oxygenation in community-acquired pneumonia: A randomized, controlled trial. Chest.

[13] Chiumello D., Brochard L., Marini J.J., Slutsky A.S., Mancebo J., Ranieri V.M., Thompson B.T., Papazian L., Schultz M.J., Amato M. (2017). Respiratory support in patients with acute respiratory distress syndrome: An expert opinion. Crit. Care.

[14] Sakuraya M., Okano H., Masuyama T., Kimata S., Hokari S. (2021). Efficacy of non-invasive and invasive respiratory management strategies in adult patients with acute hypoxaemic respiratory failure: A systematic review and network meta-analysis. Crit. Care.

[15] Ferreyro B.L., Angriman F., Munshi L., Del Sorbo L., Ferguson N.D., Rochwerg B., Ryu M.J., Saskin R., Wunsch H., da Costa B.R. (2020). Association of Noninvasive Oxygenation Strategies with All-Cause Mortality in Adults with Acute Hypoxemic Respiratory Failure: A Systematic Review and Me-ta-analysis. JAMA.

[16] Patel B.K., Wolfe K.S., Pohlman A.S., Hall J.B., Kress J.P. (2016). Effect of Noninvasive Ventilation Delivered by Helmet vs Face Mask on the Rate of Endotracheal Intubation in Patients with Acute Respiratory Distress Syndrome: A Randomized Clinical Trial. JAMA.

[17] Ferioli M., Cisternino C., Leo V., Pisani L., Palange P., Nava S. (2020). Protecting healthcare workers from SARS-CoV-2 infection: Practical indications. Eur. Respir. Rev..

[18] Ferrer M., Esquinas A., Leon M., Gonzalez G., Alarcon A., Torres A. (2003). Noninvasive ventilation in severe hypoxemic respiratory failure: A randomized clinical trial. Am. J. Respir. Crit. Care Med..

[19] Tang N., Bai H., Chen X., Gong J., Li D., Sun Z. (2020). Anticoagulant treatment is associated with decreased mortality in severe coronavirus disease 2019 patients with coagulopathy. J. Thromb. Haemost..

[20] Venables W.N., Ripley B.D. (2002). Modern Applied Statistics with S.

[21] Horby P., Lim W.S., Emberson J.R., Mafham M., Bell J.L., Linsell L., Staplin N., Brightling C., Ustianowski A., The Recovery Collaborative Group (2021). Dexamethasone in Hospitalized Patients with Covid. N. Engl. J. Med..

[22] Confalonieri M., Urbino R., Potena A., Piattella M., Parigi P., Puccio G., Della Porta R., Giorgio C., Blasi F., Umberger R. (2005). Hydrocortisone infusion for severe communi-ty-acquired pneumonia: A preliminary randomized study. Am. J. Respir. Crit. Care Med..

[23] Santus P., Radovanovic D., Saderi L., Marino P., Cogliati C., De Filippis G., Rizzi M., Franceschi E., Pini S., Giuliani F. (2020). Severity of respiratory failure at admission and in-hospital mortality in patients with COVID-19: A prospective observational multicentre study. BMJ Open.

[24] Yao Y., Cao J., Wang Q., Shi Q., Liu K., Luo Z., Chen X., Chen S., Yu K., Huang Z. (2020). D-dimer as a biomarker for disease severity and mortality in COVID-19 patients: A case control study. J. Intensiv. Care.

[25] Gattinoni L., Coppola S., Cressoni M., Busana M., Rossi S., Chiumello D. (2020). COVID-19 Does Not Lead to a “Typical” Acute Res-piratory Distress Syndrome. Am. J. Respir. Crit. Care Med..

[26] Dhont S., Derom E., Van Braeckel E., Depuydt P., Lambrecht B.N. (2020). The pathophysiology of “happy” hypoxemia in COVID. Respir. Res..

[27] Han R., Huang L., Jiang H., Dong J., Peng H., Zhang D. (2020). Early Clinical and CT Manifestations of Coronavirus Disease 2019 (COVID-19) Pneumonia. Am. J. Roentgenol..

[28] Gattinoni L., Chiumello D., Caironi P., Busana M., Romitti F., Brazzi L., Camporota L. (2020). COVID-19 pneumonia: Different respiratory treatments for different phenotypes?. Intensive Care Med..

[29] Chiumello D., Esquinas A.M., Moerer O., Terzi N. (2012). A systematic technical review of the systems for the continuous positive airway pressure. Minerva Anestesiol..

[30] Fazzini B., Page A., Pearse R., Puthucheary Z. (2021). Prone positioning for non-intubated spontaneously breathing patients with acute hypoxaemic respiratory failure: A systematic review and meta-analysis. Br. J. Anaesth..

[31] Longhini F., Bruni A., Garofalo E., Navalesi P., Grasselli G., Cosentini R., Foti G., Mattei A., Ippolito M., Accurso G. (2020). Helmet continuous positive airway pressure and prone positioning: A proposal for an early management of COVID-19 patients. Pulmonology.

[32] Radovanovic D., Rizzi M., Pini S., Saad M., Chiumello D.A., Santus P. (2020). Helmet CPAP to Treat Acute Hypoxemic Respiratory Failure in Patients with COVID-19: A Management Strategy Proposal. J. Clin. Med..

[33] Ing R.J., Bills C., Merritt G., Ragusa R., Bremner R.M., Bellia F. (2020). Role of Helmet-Delivered Noninvasive Pressure Support Ventilation in COVID-19 Patients. J. Cardiothorac. Vasc. Anesthesia.

[34] Piluso M., Scarpazza P., Oggionni E., Celeste A., Bencini S., Bernareggi M., Bonacina C., Cattaneo R., Melacini C., Raschi S. (2021). Helmet Continuous Positive Airway Pressure in COVID-19 Related Acute Respiratory Distress Syndrome in Respiratory Intermediate Care Unit. Austin J. Infect. Dis..

[35] Karagiannidis C., Hentschker C., Westhoff M., Weber-Carstens S., Janssens U., Kluge S., Pfeifer M., Spies C., Welte T., Rossaint R. (2022). Observational study of changes in utilization and outcomes in mechanical ventilation in COVID-19. PLoS ONE.

[36] Brochard L., Slutsky A., Pesenti A. (2017). Mechanical Ventilation to Minimize Progression of Lung Injury in Acute Respiratory Failure. Am. J. Respir. Crit. Care Med..

[37] Tobin M.J. (2020). Basing Respiratory Management of COVID-19 on Physiological Principles. Am. J. Respir. Crit. Care Med..

[38] Matthay M.A., Arabi Y., Arroliga A.C., Bernard G., Bersten A.D., Brochard L.J., Calfee C.S., Combes A., Daniel B.M., Ferguson N.D. (2023). A New Global Definition of Acute Respiratory Distress Syndrome. Am. J. Respir. Crit. Care Med..

