# Supplementary material for: COVID-19 Acute Respiratory Distress Syndrome: Treatment with Helmet CPAP in Respiratory Intermediate Care Unit by Pulmonologists in the Three Italian Pandemic Waves
Source: Adv Respir Med. 2023 Sep 20;91(5):383–96. doi: 10.3390/arm91050030 (PMC10514851; doi:10.3390/arm91050030)
Supplement: Supplementary file 1 [file arm-91-00030-s001.zip › arm-2574988-supplementary.pdf]

advances in respiratory medicine

MDPI

Article

COVID-19 Acute Respiratory Distress Syndrome: Treatment with Helmet CPAP in Respiratory Intermediate Care Unit by Pulmonologists in the Three Italian Pandemic Waves

Martina Piluso [1], Clarissa Ferrari [2], Silvia Pagani [1,*], Pierfranco Usai [1], Stefania Raschi [1], Luca Parachini [1], Elisa Oggionni [1], Chiara Melacini [1], Francesca D'Arcangelo [1], Roberta Cattaneo [1], Cristiano Bonacina [1], Monica Bernareggi [1], Serena Bencini [1], Marta Nadalin [3], Mara Borelli [3], Roberto Bellini [1], Maria Chiara Salandini [1] and Paolo Scarpazza [1]

[1] Lung Unit, Cardiothoracic Vascular Department Vimercate Hospital, 20871 Vimercate, Italy; martina.piluso@asst-brianza.it (M.P.); pierfranco.usai@asst-brianza.it (P.U.); stefania.raschi@asst-brianza.it (S.R.); luca.parachini@asst-brianza.it (L.P.); elisa.oggionni@asst-brianza.it (E.O.); chiara.melacini@asst-brianza.it (C.M.); francesca.darcangelo@asst-brianza.it (F.D.); roberta.cattaneo@asst-brianza.it (R.C.); cristiano.bonacina@asst-brianza.it (C.B.); monica.bernareggi@asst-brianza.it (M.B.); serena.bencini@asst-brianza.it (S.B.); roberto.bellini@asst-brianza.it (R.B.); mariachiara.salandini@asst-brianza.it (M.C.S.); paolo.scarpazza@asst-brianza.it (P.S.)

[2] Research and Clinical Trials Office, Poliambulanza Foundation Hospital, 25124 Brescia, Italy; claclafer@gmail.com

[3] School of Medicine, Surgery University of Milano Bicocca, Respiratory Unit, San Gerardo Hospital, 20900 Monza, Italy; maartanadalin@gmail.com (M.N.); maraborelli123@gmail.com (M.B.)

* Correspondence: silvia.pagani91@gmail.com; Tel.: +39-0396657090

Table S1. Complication across the three waves.

	Wave 1 n=150	Wave 2 n=180	Wave 3 n=185	Total	Chi-squared test p value
**death**	42	37	24	103	
**number of patients with Pulmonary Embolisms**	4	28	22	54	**<0.001 #**
deceased with Pulmonary Embolisms	1	7	5	13	**0.033 §**
**number of patients with thrombosis**	4	3	1	8	0.291 #
deceased with thrombosis	3	0	0	3	0.106 §
**number of patients with bleedings**	1	6	3	10	0.200 #
deceased with bleedings	0	2	1	3	0.332 §
**number of patients with supraventricular tackyarrhythmias**	4	8	3	15	0.270 #
Paroxysmal Atrial Fibrillation	2	6	3	11	
Paroxysmal Supraventricular Tachycardia	2	2	0	4	
deceased with tackyarrhythmias	1	1	1	3	0.913 §
**number of patients with pneumomediastinum**	2	7	6	15	0.368 #
deceased pneumomediastinum	1	3	4	8	0.113 §
**number of patients with pneumothoraxes**	2	1	2	5	0.760 #
deceased pneumothoraxes	1	0	0	1	0.480 §
**number of patients with severe bacterial superinfections**	0	5	7	12	0.065 #
deceased with superinfections	0	1	2	3	0.153 §
§ chi-squared test for assessing the distribution of deceased due to complication across wave deceased					

Table S2. Univariate logistic regression models with H-CPAP success (yes vs no) as dependent variable (only significant variables/predictors are reported).

Independent variables	Beta coefficient	Odds Ratio (=exp(beta))	Lower Lim OR 95% CI	Upper Lim OR 95% CI	p-value
Worst PaO_2/FiO_2 in H-CPAP (*100)	4,2987	73,6034	65,780	81,340	2,77E-45
Average FiO_2 in H-CPAP	-0,0953	0,9091	0,890	0,929	4,66E-23
d-dimer test (worst)	-0,0001	0,9999	1,000	1,000	2,81E-12
APACHE II Score	-0,1555	0,8560	0,816	0,898	1,61E-11
Lactate dehydrogenase (LDH) _ at admission	-0,0036	0,9964	0,995	0,998	4,23E-09
CARDS class_ First PaO_2/FiO_2 in H-CPAP (ref cat. Pre-CARDS)	0,8509	2,3417	1,232	4,451	1,72E-08
	-0,2936	0,7456	0,759	2,372	
	-1,1856	0,3056	0,138	0,678	
Age	-0,0509	0,9504	0,931	0,970	1,36E-07
PaO_2/FiO_2 after pronation (*100)	0,5595	1,7497	1,470	1,970	4,67E-07
PaO_2/FiO_2 in oxygen (*100)	0,7752	2,1710	1,890	2,350	7,52E-07
First PaO_2/FiO_2in H-CPAP (*100)	0,5594	1,7497	1,470	1,970	1,52E-06
STEROIDS therapy	1,5872	4,8902	2,418	9,890	5,52E-06
Interleukin-6 at admission	-0,0065	0,9935	0,990	0,997	1,10E-05
D-dimer at admission	-0,0001	0,9999	1,000	1,000	1,62E-05
Antivirals	-0,7380	0,4781	0,308	0,741	0,0011
Smoke	-0,0458	0,9552	0,364	2,509	0,0031
Hypertension	-0,5032	0,6046	0,411	0,889	0,0098
Waves wave 2 vs 1	0,3314	1,3930	0,882	2,201	0,0134
wave 3 vs 1	0,7051	2,0240	1,259	3,253	
Ischemic cardiac disease	-0,6588	0,5175	0,299	0,897	0,0208
[PaO_2/FiO_2after pronation - PaO_2/FiO_2in oxygen] (*100)	2,22E-05	1,00002	1,000003	1,00004	0,0213
Cardiovascular disease	-0,5610	0,5707	0,356	0,916	0,0219
plaquenil therapy	-0,4577	0,6327	0,421	0,951	0,0287
Average PEEP	-0,0445	0,9565	0,911	1,004	0,0354
Chronic renal failure	-0,8165	0,4420	0,203	0,964	0,0433

Table S3. Univariate Logistic regression models with Death (yes vs no) as dependent variable (only significant variables/predictors are reported).

Independent variable	Beta coefficient	Odds Ratio (=exp(beta))	Lower Lim OR 95% CI	Upper Lim OR 95% CI	p-value
Worst PaO_2/FiO_2 in CPAP (*100)	-3,6563	0,026	0,010	0,067	6,92E-28
APACHE II score	0,1966	1,217	1,150	1,288	1,34E-13
Average FiO_2 in CPAP	0,0707	1,073	1,050	1,097	5,34E-12
Age	0,0806	1,084	1,055	1,113	3,16E-11
STEROID therapy	-1,7226	0,179	0,090	0,356	1,57E-06
D-dimer at admission	0,0001	1,000	1,000	1,000	4,59E-06
Lactate dehydrogenase at admission	0,0029	1,003	1,002	1,004	7,90E-06
Interleukin-6 at admission	0,0068	1,007	1,004	1,010	1,17E-05
Smoke	-1,3171	0,268	0,035	0,284	3,73E-05
PaO_2/FiO_2 in oxygen (*100)	-0,7283	0,483	0,237	0,947	6,73E-05
CARDS class _first PaO_2/FiO_2 in CPAP	-0,7846	0,456	0,218	0,957	0,0002
Hypertension	0,7872	2,197	1,387	3,480	0,0006
ANTIVIRALS therapy	0,8548	2,351	1,456	3,796	0,0006
Cardiovascular disease	0,8997	2,459	1,481	4,081	0,0007
PaO_2/FiO_2 after pronation (*100)	-0,4264	0,653	0,390	0,844	0,0009
Ischemic cardiac disease	0,9833	2,673	1,501	4,762	0,0013

First PaO_2/FiO_2 in CPAP (*100)	-0,4034	0,668	0,325	0,899	0,0021
Wave	-0,4075	0,665	1,400	1,105	0,0026
Chronic renal failure	1,2499	3,490	1,580	7,710	0,0030
Diabetes type II (yes vs no)	0,7455	2,108	1,264	3,514	0,0055
